# Language—Mistakes—Corrections

**Published:** 1860-07

**Authors:** 


### LANGUAGE—MISTAKES—CORRECTIONS.

The English idiom requires the adjective to precede the noun.

*Hybridism* is perpetrated if languages have genera and species; in such words as "Acid Tannicum."

*Carbolic* acid may combine with albumen or gelatine forming insoluble compounds but that "*Carbonic acid*" (a gass except under immense pressure,) can combine with either of the animal tissues, but to destroy them; I have yet to learn.

And if it is so hard to learn I should like to know how Dr. F. came in possession of the belief that it might so combine and not generate mephetic and dangerous gasses.

A slight misstake on the part of a reporter who should have known better has led to a deal of learned (?) discussion upon the "Carbonic Acid" combination question. And has resulted just as all such attempts to solve the insolvable have always terminated and as far as we can see ever will terminate until we first soberly look the matter in the front and let our minds have the chance to focalize before we take sides and feel bound to maintain a position falsely taken, all for the sake of "consistency" and acting in accordance with our "better judgment."

This better judgment fallacy that so many of the blind leaders have set such high value upon is either just no judgment at all or else a peradventure pre-judging any matter which it may be proposed to exercise it upon.

All arising from a cowardly dread of running the risk of letting our fellows know (what we ourselves very well know) that as yet we do not quite know all about it, and would rather make fools of ourselves in an attempt to appear wise (hoping we *may* be right after all) in saying *something* on one side or the other of every question that is proposed. Instead of going as far as we really do know and then let the mind be without opinion upon the subject until it either gathers sufficient to form one intelligibly or gets evidence enough to constitute belief, or which is best of all, clearly perceives the demonstration so as to enable him to make it equally plain to all who have the humility to give their *attention.*

If I thought Dr. Watt capable of making the statement that is attributed to him I should be under the painful necessity of saying to him, please give some more correct chemist place, and see him with shame take a lower seat in my regards and real merit.

It may be well that this thing has "been in blessing sent us." And I hope it may prove the folly of our own bowing to authority irrespective of close scrutiny and truthfulness to principle.

As long as naked authority sways the mind we shall be pinning our faith to some one else's sleeve, and in all time of trial must have resort to our fountain of strength in the repository of authority, whether in books, persons or modes, or utterly fail to effect (except by the merest chance) the highest good to those for whom we operate.

If I or some other one not learned in chemistry had made the statement it would either not have seen the light or if it did would have been corrected beforehand.

But coming as it did from one to whom we looked up with a confidence it was *misunderstood*, and in accordance with due respect and proper regard for authority a mere misstatement supplanted the real statement, and so an error crept in at the same hole that all falsities and negations ever do.

This folly of mistaking an "ignis-fatuus" for the orb of day, because it was in a line with the "orient" is no new thing under the sun. And I do hope this lesson, with many others like it, originating *near* the source of this, may teach us that it matters not how clearly defined a proposition may be in the mind of him who enunciates it, it is no demonstration to the learner until he has taken in the necessary perception of the position to render it *past* opinion or belief by becoming a matter of *knowledge* to him.

The reporter may be excused on the ground of his being occupied more with *sounds* than *ideas.* But that a well posted man could deliberately entertain such lucubrations as are evident upon the reading, must have occupied him when writing. I acknowledge my inability to make tally with consistency. "SEARCHER."

We are at a loss to know what Searcher means by the above.—ED.

